# Primitive pelvic bone hydatidosis: What an amazing extension

**DOI:** 10.1002/ccr3.5054

**Published:** 2021-12-11

**Authors:** Soumaya Boussaid, Cyrine Daldoul, Maroua Hassayoun, Sonia Rekik, Samia Jammali, Hela Sahli, Mohamed Elleuch

**Affiliations:** ^1^ Rheumatology Department Rabta Hospital Tunis Tunisia; ^2^ Faculty of Medicine of Tunis University Tunis el Manar Tunis Tunisia

**Keywords:** echinococcosis, hip, hydatid disease, pelvis

## Abstract

Hydatidosis is an anthropozoonosis mainly encountered in pastoral areas. It mostly affects the liver, lung, and rarely the bone and the soft tissues. Skeletal involvement is usually secondary to visceral hydatidosis. We report a case of a 49‐year‐old man presenting with one‐year history of a progressive left hip pain. On local examination, there was tenderness in the left gluteal region with reduction in the hip range of motion. Pelvic X‐ray revealed an expansive bone destruction involving the left hemi pelvis without periosteal reaction. A magnetic resonance imaging showed multiple cystic lesions extending from pelvic bones to the gluteal region. The possibility of hydatid disease was raised, and hydatid serology test was positive. No visceral involvement was found by additional examinations investigations revealed visceral hydatidosis. Thus, the diagnosis of a primary bone hydatid disease was established. No surgical excision was possible, and the patient was put on Albendazole. Echinococcosis should be ruled out while dealing with progressive expansive bony lesions. Surgical management remains a challenge especially if the involvement is very extensive.

## Graphical abstract

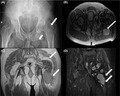

## 1 INTRODUCTION

Hydatidosis is an anthropozoonosis caused by the larval stage of Echinococcus tapeworms.^1^ It is a worldwide infectious disease but highly endemic in North Africa, the Middle East, and South America.^2^ It could be caused by different species, but Echinococcus granulosus species is responsible for more than 95% of human hydatid disease.^3^ The latter is mainly encountered in pastoral areas where the contact between dogs and sheep is frequent. The prevalence of this disease is most likely underestimated due to its asymptomatic character.^3^ This parasitic disease most commonly affects the liver and the lung; however, it seldom concerns the bone and the soft tissues. In fact, the musculoskeletal involvement in hydatid disease is only reported in 0.5 to 2% of all cases and it is usually secondary to visceral involvement.^1^, ^4^ Although it is an old disease, the diagnostic of hydatid bone is still challenging given the delayed clinical manifestations and the nonspecific radiological features.^5^

We herein report a case of a primary bone hydatid disease of hip, pelvis, and the surrounding soft tissues.

## 2 CASE REPORT

A 49‐year‐old man was admitted to our hospital with one‐year history of a progressive left hip pain that is restricting his daily activities. The patient was living in an urban area with no significant past medical history. He did not report fever, chills, or loss of weight. There was no history of tuberculosis, no prior trauma, and no contact with animals. The patient was limping and walking with the help of crutches. On local examination, there were no signs of skin inflammation. There was tenderness in the left gluteal region, and the mobilization of the left hip was painful with reduction in the hip range of motion.

Routine blood investigation did not reveal an elevated leucocyte count nor eosinophilia. C‐reactive protein and erythrocyte sedimentation rate were within normal range.

**FIGURE 1:**
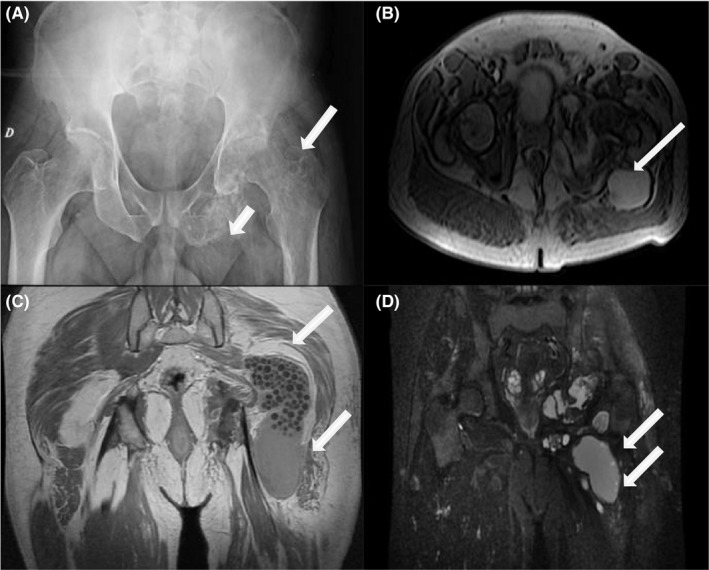
(A) Pelvic X‐ray showing osteolytic lesions in the ischiopubic branch and all the left femur and proximal extremity of the tibia. (B) MRI of the pelvis: axial section in T1 gado sequence sowing multilocular appearance of the femoral head with breach of the bone cortex of the femur with a very large intra‐articular effusion. (C) Coronal section in T1 gado sequence with Fat Sat sowing involvement of the ischial branch of the pubis and a very large intra‐articular effusion and multilocular appearance of the soft parts. (D) Coronal section in T1 sequence sowing multilocular appearance of the soft parts

Pelvic X‐ray revealed an expansive bone destruction involving the left ischium, ilium, acetabulum, femoral head, and the trochanteric region without periosteal reaction (Figure 1A). Hip ultrasound showed an intra‐articular effusion without collection. A magnetic resonance imaging (MRI) was performed and showed multiple cystic lesions extending from the ischium and ilium to the trochanteric region with cortical bone destruction. The bone lesions were associated with a multiloculated cystic mass in the gluteal region. The cysts showed intermediate or low signal intensity on T1‐weighted images and hyperintensity on T2‐weighted images in the involved bone and the surrounding soft tissue (Figure 1B‐D).

Based on imaging findings, the possibility of hydatid disease was raised and hydatid serological tests (the enzyme‐linked immunosorbent assay “ELISA” and the immunoblot test) were then performed. They both showed positive results. Clinical examination, and chest and abdominal imaging did not reveal any collection or mass in the liver, lungs, or any other viscera. Thus, the diagnosis of a primary bone hydatid disease was established.

The patient was put on Albendazole by oral administration, and he was proposed for surgery but refused by the patient.

## 3 DISCUSSION

Echinococcosis granulosus has definitive hosts dogs and other carnivores where the adult tapeworm lives and those hosts are responsible for the dissemination of the parasite's eggs. The eggs can contaminate intermediate hosts which are sheep and other herbivores and can also contaminate human beings who are considered as incidental hosts.^3^ Hydatid disease occurs through the fecal oral route after the ingestion of contaminated vegetables or water.^6^ The parasite passes the intestinal wall and gains the portal venous system explaining why the liver is the most common involved organ.^1^ Then, it spreads throughout the body and it can be found almost in every part of the body.^7^

However, bone involvement remains rare accounting for less than 2%. The infestation is primarily via hematogenous seeding, and usually spongy vascularized bones are the most common site of osseous hydatidosis like vertebrae (50%), pelvis (21%), and femur (16%).^8^, ^9^ When located in the bone marrow cavity, hydatid lesions grow slowly; they can spread along the trabecular bone and cause bony destruction.^5^ They can penetrate through the bone cortex and overrun the surrounding soft tissue. Unlike other viscera, there is no adventitia formation in skeletal hydatidosis; thus, daughter cysts can cause microvesicular invasion into the bony tissue.^9^, ^10^ As a result, complete eradication of the parasite is often unsuccessful despite extensive surgery and hence those sites are considered as severe ones. Intramuscular hydatid is rare, and it occurs in 0.7 to 0.9% of patients. This is due to the difficulty of cyst growth in the presence of muscle contraction and lactate‐containing environment.^3^, ^11^ There might be a slight tendency in the muscle of proximal extremities due to more vascularity and fewer movements.^3^

Hydatid bone disease can be a challenging diagnosis as symptoms are nonspecific.^12^ In fact, they depend on the size of the lesion, the host immune response, and the existence of complications such as infection and cyst rupture.^3^ In our case, the extension of the hydatidosis is very important, and to our knowledge, there is no such important extension which has been reported in the literature.

The most frequent symptoms are pain, local swelling, and fracture.^1^ It must be emphasized that those symptoms have significant overlap with bone malignancies but the evolution of hydatid disease is slower and it is not associated with inflection of the general condition or fever.^3^, ^13^ In case of pelvic involvement, the main complaint is usually pain and swelling in the gluteal region accompanied by reduction in the hip range of motion.^9^ So was the case of our patient.

Bone hydatidosis was reported to be more prevalent in men with an average age of 50 years.^13^ Epidemiological arguments might help the diagnosis as history of pastoral contact seems to be an important risk factor.^3^, ^5^ In addition, a relatively higher prevalence of hydatid disease was noted in the rural populations and that is mainly attributable to the slaughtering of animals in these areas.^5^ However, those elements are not always present, as it is in our case, and their absence should not misguide the diagnosis.

The main basis for diagnosis is still radiological imaging including ultrasonography, plain radiographs, and CT scans with MRI considered as the best imaging modality when soft tissues are involved.^14^ However, the first‐line tool is conventional radiographs.^3^ It mostly does not show specific features. The common picture is irregular osteolysis with grouping of cystic lesions giving a “waffle” like appearance as described in our case.^9^ Usually, the lesions do not include periosteal reaction nor peripheral calcifications.^12^ Ultrasound is considered as a cost‐effective imaging modality for soft tissue masses. It may reveal a water attenuation cyst with a well‐defined wall encircling floating membrane, hydatid sands, and vesicles, but most of the time it is not accurate for intramuscular echinococcosis diagnosis.^14^ It is more suitable for follow‐up after treatment.^3^ Computed tomography (CT) is particularly useful for the evaluation of osseous involvement and for the detection of eventual calcifications in the cysts.^3^ The intralesional calcifications should be looked for as they are highly suggestive of hydatid bone disease and help in the differential diagnosis.^5^ However, they are rare and they are more frequent in the extraosseous parts.^15^ In pelvic bones, CT scans typically reveal single or multiple hypodense lesions with internal septa. Bone expansion is accompanied by cortical thinning and destruction and does not show periosteal reaction unlike other bone tumors.^3^, ^5^, ^13^ It can show soft tissues masses mimicking tuberculous abscesses but still less performing than MRI.^5^, ^13^ On MRI, hydatid cysts in bone and soft tissues have an intermediate or low signal in T1 sequence and high signal intensity in T2 sequence.^5^ The lesions are not enhanced while gadolinium injection. Detached membrane from cyst wall appears hypointense on all sequences (serpent sign), and it can be enhanced after gadolinium injection.^15^ The outer layer of the cyst—if it exists—represents pericyst which appears hypointense on T2‐weighted images (rim sign).^15^ The lesions can be single or polycystic, but extraosseous lesions are often single cystic, thick‐walled, and spherical due to the low resistance of the soft tissues.^13^

In the absence of elements pointing toward echinococcosis in the patient's history, these radiological features often cause misdiagnoses. Imaging differentials include neoplastic lesions, bone metastasis, giant cell tumor, aneurysmal bone cyst, and chronic osteomyelitis tuberculousis of the bone. Solitary lesions can mimic solitary bone cyst, plasmocytoma, and brown tumor of hyperparathyroidism, while chronic hydatidosis can mimic fibrous dysplasia.^4^, ^15^

Those imaging findings might be combined with serological tests to make a more accurate assessment of the disease.^14^ In fact, a great number of patients with bone hydatidosis represent a positive serology as the lack of pericyst in bone facilitates the release of antigens.^1^, ^3^ Different serological tests are available. The chief serological tests carried out include the enzyme‐linked immunosorbent assay (ELISA), indirect hemagglutination test (IHA), and the immunoblot test (IB) to detect specific antibodies and circulating antigens. While ELISA is more sensitive, the IB techniques are more specific.^16^ Therefore, many have suggested using a combination of both during the serological diagnosis of cystic hydatidosis. However, despite recent advances, these tests are of limited value when the hydatid cyst is aging, calcified, or dead.^5^

It is challenging not only to diagnose echinococcosis of bone but also to treat the lesions. The management of osseous hydatidosis is often complicated as the disease is discovered at the late stage.^11^ The recommended therapy for osseous and extraosseous cysts in hip and pelvis is an extensive complete surgical excision.^1^ In the published medical literature, several surgical methods, including simple curettage and aspiration, complete excision to hemipelvectomy, and replacement of bone defects with total hip arthroplasty, bone grafting, have been reported thus far.^5^ Surgical techniques are generally combined with pre‐ and postoperative chemotherapy to prevent recurrence; however, there is no consensus on the dose and the duration.^1^, ^11^, ^14^ Albendazole is regarded as the first‐line chemotherapy in the treatment of bone lesions, because of its higher blood plasma levels.^1^ In some cases where the surgery is a risky option, Albendazole alone can be proposed but with high risk of extension and recurrence.^12^

Even with the best of efforts in treatment, pelvic hydatidosis carries grave prognosis that it has been called “white cancer”.^11^ In fact, it is associated with high rate of recurrence of up to 50% and with poor functional outcomes in the long run.^1^, ^9^

## 4 CONCLUSION

In areas where hydatid disease is endemic, the diagnosis of pelvic echinococcosis should be kept in mind in case of any bone lesion. We reported this case in order to raise awareness about the nonspecific features of this disease and the importance of adequate radiological and serological investigations to establish the right diagnosis as soon as possible. Surgical management remains a challenge, especially if the involvement is very extensive.

## CONSNET

Published with the written informed consent of the patient.

## Data Availability

The datasets used and/or analyzed during the current study are available from the corresponding author on reasonable request.
